# Nominations and Places of Meeting

**Published:** 1867-06

**Authors:** 


NOMINATIONS AND PLACES OF MEETING.

The Committee on nomination of officers, and place of meeting, reported as follows:

Place of meeting, New Orleans.

*President*—Samuel D. Gross, of Pennsylvania.

*Vice-Presidents*—A. C. Post, of New York; Jno. H. Atlee, of Pennsylvania; D. W. Yandell, of Kentucky, and H. R. Storer, of Massachusetts.

*Permanent Secretary*—William B. Atkinson, of Pennsylvania.

*Assistant Secretary*—J. G. Richardson, of New Orleans.

*Treasurer*—Casper Wistar, of Pennsylvania.

On motion of Mr. Davis, of Illinois, that portion of the report, naming the place of meeting and officers resident there, was laid on the table. The rest of the report was then adopted.

Dr. Davis remarked that the crisis had arrived, which he had long anticipated, when the matter of eating and drinking, and entertaining the Association had come to involve such an expense, that no invitation had been extended in advance, by resident members of the profession, for the Association to meet in any city, and the Committee had reported New Orleans, without an invitation from any one there. While there was no city in which he would like better to meet on personal accounts, and as a manifestation of reunion with the south, he felt that it would not be right to impose a meeting of the Association on that city. It was embarrassed in every relation of life, like all other places in that direction. It was impoverished, and though they would no doubt receive us with cordial and open hands, it would be wrong to tax them in that way. They could not vie with the liberality and extravagance of Cincinnati; or if they did it would be at a sacrifice we should not admit of. He would give them another year to recover, and an opportunity to invite us. He therefore offered the following:

*Resolved*, That the next annual meeting of the American Medical Association shall be held in the City of Washington, on the first Tuesday in May, 1868, and every second year thereafter, until otherwise ordered by the Association.

*Resolved*, That whenever the Association shall meet in the City of Washington, as directed in the above resolution, the Committee of Arrangements be strictly forbidden either to provide themselves, or except provision by others, of any entertainment or excursion whatever.

Dr. Yandell, of Ky., arose, and was urged to take the stand, where he said: "I have listened with pleasure, and derived some new light from the remarks of the gentleman from Chicago. I arise to explain the motives of the Committee in naming New Orleans, and in doing which, I think I shall violate no confidence. When the place of meeting was called for, Dr. Horatio Storer of Mass., proposed New Orleans. The motion was seconded by Dr. Alonzo Palmer of Mich. As one of the few representatives from the South, I chanced to be in the Committee, and was asked my opinion in reference to New Orleans. I confess to you, gentleman, that I never listened to a proposition in all my life which excited such emotions in my breast, as this motion coming from Massachusetts, and seconded by Michigan, to take this great body, of brethren across the crimsoned waste of war, and hold out the hand of fellowship to their brethren in the South. [Cheers.] I confess to you that when Dr. Storer, of New England, and Dr. Palmer, of the mighty west, moved that we thus extend the hand of brotherhood to the medical profession in the South, I could not restrain my emotions from bringing tears of joy to my eyes.

The first question was whether it would be acceptable to New Orleans. I felt that I had some right to speak for the profession in New Orleans. My excellent father and myself had taught many of them as students in medicine. I had served with them, and was personally acquainted with most of those who had been in the Southern army. I knew they felt as I feel, that when peace came in 1865, we had been united again; in fact, that we had never been divided; that though politicians had separated us by geographical lines, still the great republic of letters was one; that

"No pent up Utica contracts our powers,
But the whole boundless continent is ours!"

In the great republic of science all geographical lines should be obliterated. We of the South, and you of the North, the East, and the west, have a common heritage. The ashes of the illustrious Drake lie in your beautiful cemetery here. Have we of the South no claim to, nor interest in his name and his fame? Heve you of the North no interest in the long list of great and good men who adorn the profession in the South? No interest in those whose labors have added glory not only to medicine but to the nation?

Hence I said I believed every man in the South, in that land of flowers, of the mocking bird, and of beautiful women, waited but to have your hands extended to them to give you a welcome. [Cheers.] I said to the Committee that New Orleans was poor, that the whole South was poor, but that you would not be less welcome to sit under our vines, and to take fruit from our fig trees; that we could not give you such splendid entertainments as Cincinnati has done; but we could do what was better than eating and better than drinking, we could give you the warm grasp of the hand that you would take with you in memory to your homes. In view of this the Committee honored itself and honored New Orleans by selecting that place for its next meeting.

I see the objection of Dr. Davis, but if he proposes to do no eating and no drinking at the next meeting, New Orleans is a better place than Washington City. [Applause.] If you want to put the Medical Association on low diet, go down there. [Cheers.] But the very reason of all others which should take the Association South of Mason and Dixon's line—if such a line there be now—is that the Southern people are not able to come to you. They are not here to-day because they could not afford to come. But they would welcome you to their homes. If you do not go to New Orleans, then go to some place within their reach.

Dr. Griswold, of Ohio, moved to substilute Knoxville, Tenn., instead of Washington City.

Dr. Sayre moved to substitute New Orleans, as in the original report. He thought that with Dr. Davis' resolution of restriction as to entertainments, there would be no objection to that place.

Dr. Cox, of Md., indorsed all that had been said by his friend from Kentucky; was proud that one man from the South had expressed himself as standing on the platform of the union of the medical profession. He never supposed there would be any difficulty in reuniting the profession of medicine after the war was over. As to the entertainments of the Association, he was in favor of them, and thought if Dr. Davis' measure was adopted the usefulness and attendance of the Association would be impaired. In England they did far more eating and drinking than was done here, and yet they accomplished a large amount of work.

Dr. Storer, of Boston, said the object of these meetings was to bring as many of the profession together as possible, and it should meet in such places as would accomplish that object. We should not meet where we have not been invited by resident members. That thing had been tried in Boston, and came near failing. The probability was we would be well received; but we ought to wait for a proper invitation. The hour having arrived for the steamboat excursion, the whole subject was laid upon the table.

